# Streptomyces spp. in Arid and Savannah Ecosystems: Effects on the Inhibition of Actinomycetoma Pathogens

**DOI:** 10.7759/cureus.101153

**Published:** 2026-01-09

**Authors:** Mohamed E Hamid, Martin R Joseph, Ahmed B Abd Alla, Mogahid M El Hassan

**Affiliations:** 1 Department of Clinical Microbiology and Parasitology, College of Medicine, King Khalid University, Abha, SAU; 2 Department of Clinical Microbiology and Parasitology, King Khalid University, Abha, SAU; 3 Department of Parasitology and Medical Entomology, College of Medical Laboratory Science, Sudan University of Science and Technology, Khartoum, SDN; 4 College of Medical Laboratory Science, Sudan University of Science and Technology, Khartoum, SDN

**Keywords:** 16s rrna gene bioassays, actinomycetoma, biodiversity, ecosystems, inhibitory effects, soil properties, s. sudanensis, streptomyces spp

## Abstract

Background

Knowledge of the ecological and soil differences between arid and savannah ecosystems is essential for assessing their biodiversity and potential for agricultural and disease-controlling applications. Arid zones are characterized by extreme temperatures and limited rainfall, supporting unique soil types that influence microbial communities. This study examined soil *Streptomyces* spp., known for their antibiotic properties, focusing on their inhibitory effects against *Streptomyces sudanensis*, a pathogen associated with actinomycetoma.

Methodology

Soil samples (n = 7) were collected, and physicochemical parameters, along with enzyme activities, were analyzed. *Streptomyces* spp. were isolated, characterized morphologically and phenotypically, and identified molecularly via 16S rRNA gene sequencing. Their inhibitory effects against *S. sudanensis* were evaluated using agar-based bioassays.

Results

*Streptomyces *spp. (n = 64), including *Streptomyces griseostramineus*, were identified. The results indicated significant ecological differences: arid sites had high temperatures (29.7°C) and low rainfall (70 mm) with nutrient-poor Yermosols, whereas savannah sites had higher rainfall (501 mm) and nutrient-rich Arenosols and Vertisols. Inhibition zones varied significantly, with arid ecosystem isolates showing a higher mean inhibition value (2.8411) compared to savannah isolates (1.7139). Statistical analysis verified a significant difference in mean inhibition (t = 2.589, p = 0.0126), suggesting distinct inhibitory capacities linked to the ecosystem.

Conclusions

This study emphasizes the significant ecological and soil differences between the ecosystems and proves that soil *Streptomyces* spp. in arid regions possess distinctly higher inhibition potential against *S. sudanensis*, suggesting their potential as biological agents in these environments.

## Introduction

*Streptomycetes *are high-G + C Gram-positive, spore-forming bacteria belonging to the *Streptomycetaceae *(order *Actinomycetales*), which includes more than 500 species [1]. These bacteria are generally distributed in soil and are more abundant than other soil bacterial genera. Soils are considered reservoirs for streptomycetes, including the few pathogenic species, and their propagules, with transmission to humans often occurring through contact with the sharp thorns of *Acacia* trees, which can carry streptomycete spores [2].

*Streptomyces* spp. are well known for their diverse metabolic capabilities, particularly their production of secondary metabolites with antimicrobial properties. They are widely distributed in soil ecosystems and play a crucial role in nutrient cycling and organic matter decomposition [3,4]. Soil characteristics, such as nutrient composition, moisture, and pH, significantly influence microbial community structure and function. Understanding these relationships is critical for harnessing the potential of *Streptomyces* as biocontrol agents. Actinomycetoma is a chronic granulomatous disease primarily caused by various microorganisms, with *Streptomyces somaliensis* and *Streptomyces sudanensis* being the most significant pathogens [5]. This condition affects the skin, subcutaneous tissues, and, in some cases, the bones, leading to the formation of tumor-like lesions. If left untreated, actinomycetoma can result in severe morbidity, including significant deformities and disability [6]. The prevalence of actinomycetoma in this region, mainly Central Sudan, particularly in Gezira State, Sennar, and the White Nile, has reported cases, especially in rural areas such as Sennar, North Kordofan, and the White Nile. Environmental conditions in these regions promote fungal growth and transmission, particularly linked to agricultural activities and walking barefoot, drawing attention to the need for effective management strategies. Recent studies suggest that exploring natural antagonistic agents present in the environment may provide new avenues for treatment and disease control [7].

Current research has focused heavily on the ecological roles of *Streptomyces*, but a critical knowledge gap exists concerning how soil properties influence their pathogenicity, especially in relation to actinomycetoma. Our research explores the link between soil properties and *Streptomyces* spp. distribution in arid and savannah ecosystems. We aim to assess their diversity, evaluate their inhibitory activity against *S. sudanensis* (an actinomycetoma pathogen), and compare isolates to known mycetoma strains. This work seeks to highlight *Streptomyces *spp. as promising biological agents for soil health and pathogen control.

## Materials and methods

Soil sampling

**Figure 1:**
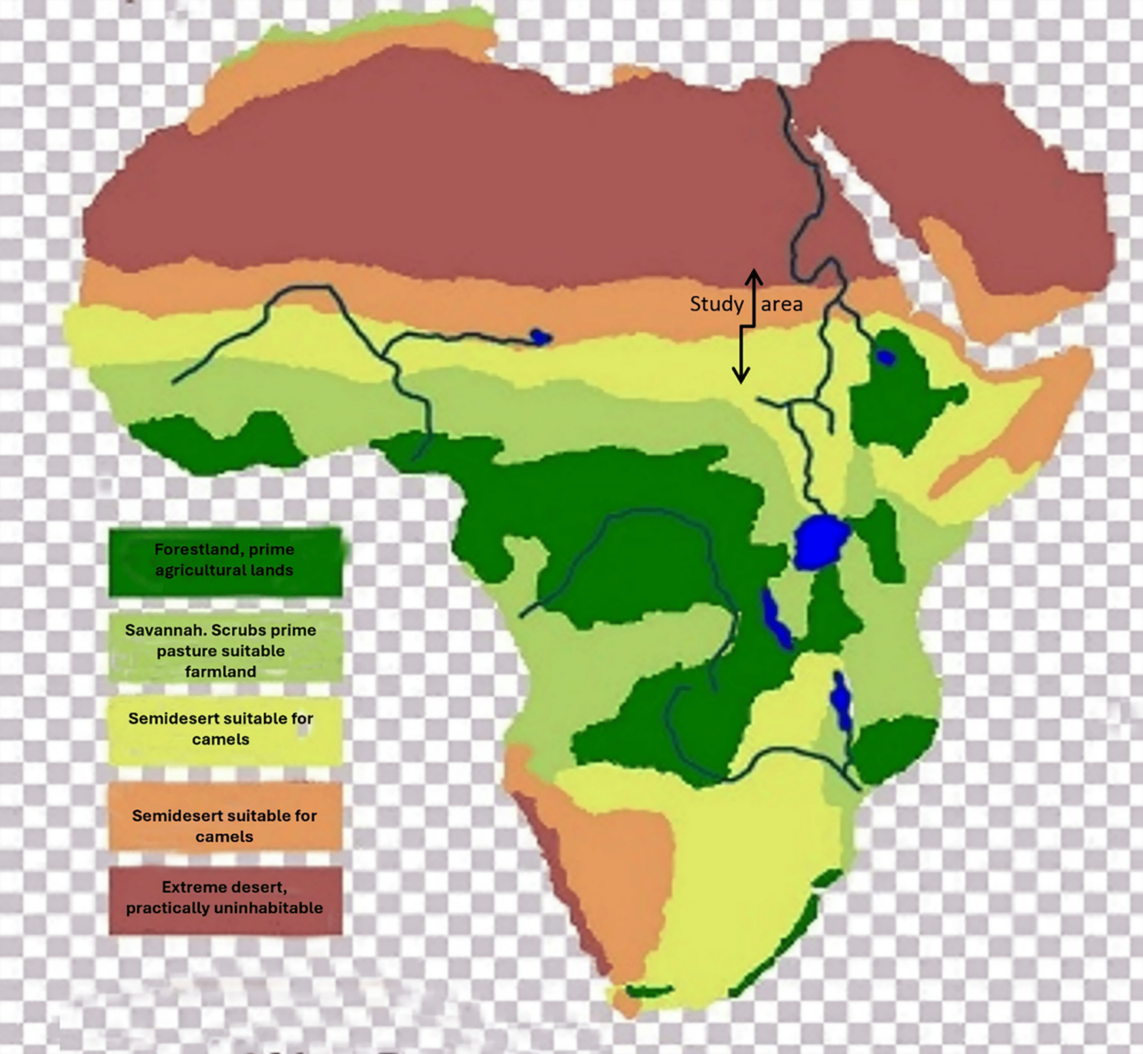
Map of African savanna grassland geography, highlighting the study area within the geographical borders of Sudan. Originally sourced from imgbin.com.

Soil samples were collected from multiple sites within arid and savannah ecosystems during the dry season. This timing was important as it reflected the natural (hot and dry, Figure 1) conditions under which *Streptomyces* spp. survive. Soil samples (merging four subsamples from each site) were collected at a consistent depth of ~5 cm to ensure comparability, specifically targeting the surface microbiome, such as *Streptomyces*. This depth was chosen because the surface habitat is influenced by the ecosystem and can affect hosts, including humans, animals, and plants.

Soil physicochemical parameters and enzyme assays were conducted, as described before [7]. Soil samples were analyzed for soil type, average annual temperature (°C), average annual rainfall (mm), phosphorus (P) (mg/100 g), potassium (K) (mg/100 g), soil pH, and enzyme activities (N-acetylglucosaminidase, glucosidase, cellulase, phosphatase, xylanase in nmol/g soil). Moisture content was determined by drying and weighing, while enzyme activities were assessed using soil extracts and spectrophotometric techniques, expressed as activity per gram of soil to evaluate soil health and microbial activity [8].

Isolation of *Streptomyces *spp.

*Streptomyces* were isolated using high nitrogen content (HNC) medium (6% yeast extract, 0.05% SDS, 0.05% CaCl_2_, pH 7.0). Soil (0.5 g dry weight) was added to the HNC medium and incubated at 120 rpm and 42°C for one hour. The suspension was allowed to settle, and dilutions (1:5, 1:10, 1:30) were spread onto ISP2 and humic acid agar plates, with ISP2 supplemented with antibiotics to prevent contamination. Plates were incubated at 27°C for up to three weeks. Colonies with typical morphology were streaked on ISP2 for purification and stored at −20°C in 20% glycerol soil, as described previously [7].

Morphological examination

The morphological characteristics of the isolates were examined using light microscopy to assess colony morphology, spore formation, and other distinguishing features, as detailed previously [1]. This analysis involved assessing various features, including colony morphology, cellular shapes, and arrangements. Strains that exhibited similar characteristics were clustered together, resulting in the formation of preliminary *Streptomyces *color categories. These groupings will provide a foundation for more thorough investigations, enabling researchers to delve deeper into the diversity and potential applications of these isolates.

Molecular identification of soil isolates

The 16S rRNA gene was amplified using universal primers 27F and 1492R, following Rainey et al.’s method [9]. Amplification (20 μL) was performed with Promega Green Mix, and products were analyzed via 1% agarose gel electrophoresis on a Bio-Rad MyCycler.

For sequencing, chromosomal DNA was isolated from a seven-day culture using PEG 200. The same primers were used for amplification and sequencing, analyzed with the ABI 370XL DNA analyzer and SeqMan software.

DNA sequences were corrected with Chromas and aligned with 16S rRNA sequences in the EZbioCloud database to evaluate phylogenetic relationships. A phylogenetic tree was constructed using the MEGA X program with the Neighbor-Joining method and 100 bootstrap replications.

Inhibitory effects against *Streptomyces sudanensis*

The inhibition of *S. sudanensis* was evaluated using an agar-based bioassay, as described previously [7]. Fresh suspensions (0.1 mL) of *Streptomyces* isolates were placed on ISP2 agar plates previously streaked with *S. sudanensis* (DSM 41923). Multiple *Streptomyces* isolates were cultured per plate. The inhibition activities were calculated as the ratio between the inhibition zone (mm) against *S. sudanensis* and the colony size (mm) of soil streptomycetes. Inhibition zone/colony diameter (mm) provides a standardized measure of antimicrobial activity independent of the isolate’s colony size.

Statistical analysis

Data were analyzed using paired samples tests in PAST (Version 5.0.2; Hammer Ø, Harper DAT, & Ryan PD, Natural History Museum, University of Oslo, 1999-2024) to compare mean inhibition values between the arid and savannah ecosystems, calculating the mean difference and 95% confidence intervals. A t-test assessed the significance of the mean differences.

## Results

Table 1 presents an overview of soil factors and environmental conditions across various sites in arid and savannah ecosystems. The data includes site names, coordinates, ecoregions, and soil types, alongside key environmental metrics such as average annual temperature and rainfall.

**Table 1:** Soil factors and environmental conditions in arid and savannah ecosystems. NAG = N-acetylglucosaminidase; GLU = β-glucosidase; CEL = cellulase; PHO = phosphatase; XYL = β-xylosidase

Site code	Site name (coordinates)	Ecoregion	Soil type (common description)	Average annual temperature (°C)	Average annual rain (mm)	P (mg/100 g)	K (mg/100 g)	Soil pH	NAG activity (nmol/ hour.−1 g soil^− 1^)	GLU activity (nmol/ hour.− 1 g soil^− 1^)	CEL activity (nmol/ hour.− 1 g soil^− 1^)	PHO activity (nmol/ hour.− 1 g soil^− 1^)	XYL activity (nmol/ hour.− 1 g soil^− 1^)
7	Hussein Narti-1 (18.0299° N, 31.4301° E)	Arid	Yermosol (Desert sand)	29.7	70	4.6	32.6	8	2.2	4.1	-	1.9	0.2
19	Hussein Narti -2 (17.7861° N, 31.3137° E)	Arid	Yermosols (desert sand)	29.7	70	5.3	38.3	8	2.4	4.9	-	-	0.2
8	El Muglad (11.0347° N; 27.7491° E)	Savanna	Arenosols (stabilized sand dunes with silt or clay)	28.5	501	2	28.2	6.4	25.8	54.3	5	85	8.4
10	Nyala (12.0518° N 24.8805° E)	Savanna	Arenosols (stabilized sand dunes with silt or clay)	27.2	398	1.5	19.2	7.1	9.5	19.3	-	27	1.9
14	Umm Ruwaba (12.9028° N; 31.2283° E)	Savanna	Arenosols (stabilized sand dunes with silt or clay)	27	375	6.7	22.2	6.8	0.7	9.9	-	24	0.2
23	Al Fashir (13.6198° N; 25.3549° E)	Savanna	Arenosols (stabilized sand dunes with silt or clay)	26	213	28.6	60	7.7	4	12.1	0.5	13.6	1.2
29	Ad Damazin (11.7855° N; 34.3421° E)	Savanna	Vertisols (black clay)	28.3	713	16.5	49.1	7.3	18.9	87.2	3.4	80.5	8.7

Arid sites (Hussein Narti-1 and Hussein Narti-2) exhibit high temperatures (29.7°C) and low rainfall (70 mm), with Yermosol soil types showing limited nutrient availability and low enzyme activity. In contrast, savannah sites, such as El Muglad and Nyala, demonstrate greater rainfall (up to 501 mm), slightly lower temperatures, and a variety of Arenosols and Vertisols. These savannah soils exhibit higher nutrient concentrations and enzyme activities, indicating a more dynamic and fertile environment compared to the arid regions. Overall, the results highlight significant differences in soil characteristics and environmental conditions between arid and savannah ecosystems.

**Figure 2:**
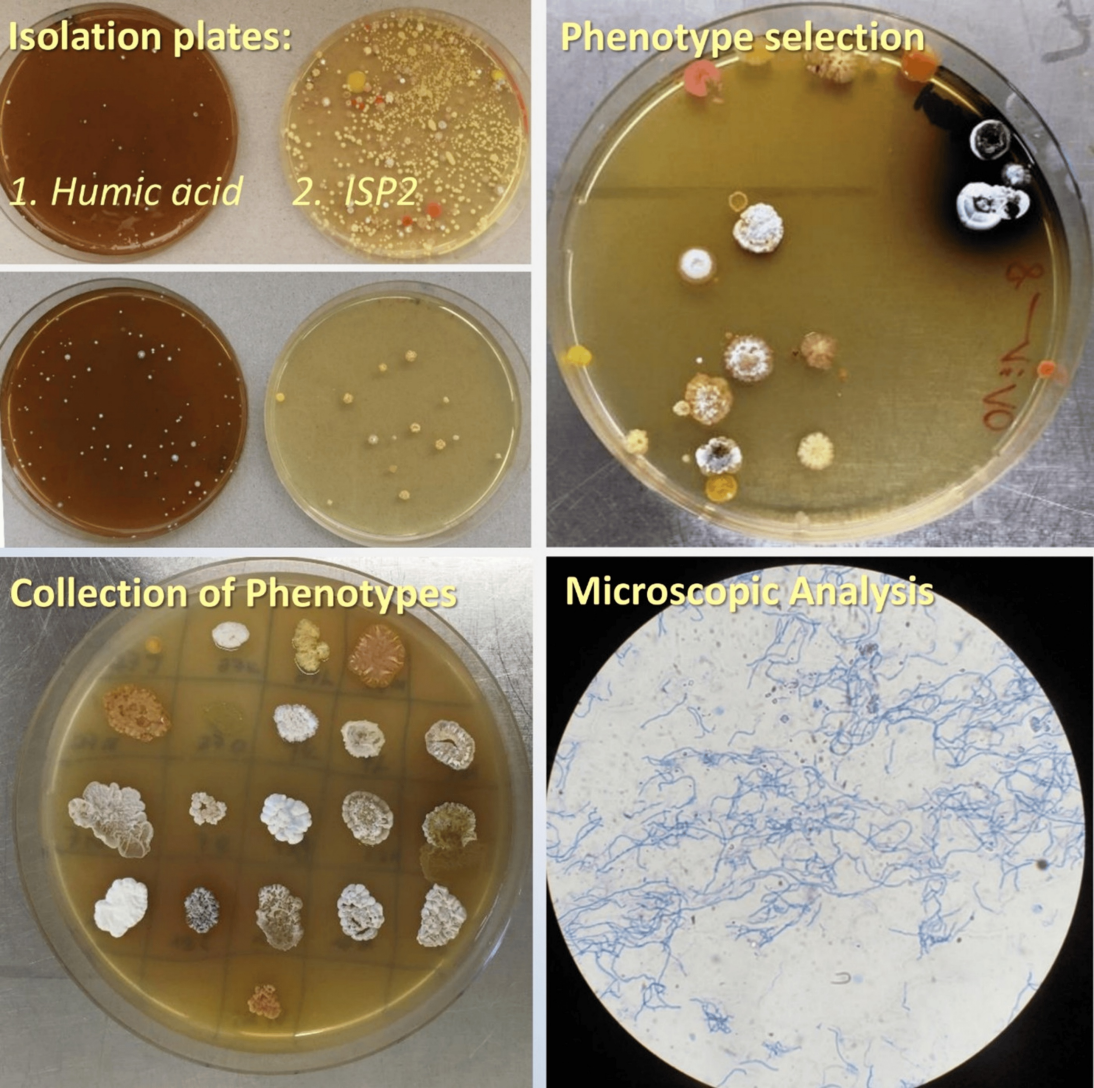
Isolation, selection of unique phenotypes, and microscopic analysis of Streptomyces species recovered from soil samples.

A total of 64 strains were identified, predominantly belonging to the genus *Streptomyces*. Notable species among these include *Streptomyces griseostramineus*, *Streptomyces albogriseolus*, and *Streptomyces werraensis*. The isolation was achieved using humic acid and ISP2 media. Figure 2 illustrates the phenotype selection process, purification, and microscopic analysis. A remarkable variety of phenotypic variations is presented, along with microscopic images of their distinctive filamentous structures.

Table 2 summarizes the identification of various soil *Streptomyces* strains from arid and savannah ecosystems, detailing their similarity percentages to closely related species and corresponding GenBank accession numbers. This overview is based on 16S rRNA sequencing and provides insights into each strain’s closest species and the percentage of nucleotide relatedness. The inclusion of GenBank accession numbers facilitates further research and verification of the sequences.

**Table 2:** Summary of soil Streptomyces spp., their identification to the closest species based on 16S rRNA sequences, their GenBank accession numbers, and average inhibition values against Streptomyces sudanensis. *: Inhibition zone/colony diameter (mm) provides a standardized measure of antimicrobial activity independent of the isolate’s colony size; **: NA, not available, has been excluded due to incomplete sequence length and poor quality.

Strain code	Identification	Identification and % nucleotide relatedness	Gb_ Accession	Inhibition zone (inhibition/biomass diameter, mm)*
7A	*Streptomyces *spp.	97.00% with *S. griseostramineus*	MF356350	0
7B	Streptomyces griseostramineus	99.89% with *S. griseostramineus*, 99.89% with *S. griseomycini*, and 99.89% with *Streptomyces* spp. SD528 (EU544233)	MF353983	4
7C	*Streptomyces* spp.	97.63% with *S. chromofuscus*	MF356351	2.33
7D	*Streptomyces *spp.	99.33% with *S. levis*	MF356352	6.25
7E	*Streptomyces *spp.	98.80% with *S. albaduncus*	MF356353	5.3
7F	Streptomyces prasinosporus	99.90% with *S. prasinosporus*	MF353984	3.5
7G	*Streptomyces* spp.	98.76% with *S. minutiscleroticus*	MF356354	3
7H	Streptomyces leeuwenhoekii	99.77% with *S. leeuwenhoekii*	MF353985	2.5
7I	*Streptomyces *spp.	99.08% with *S. albaduncus*	MF356355	2.5
7J	*Streptomyces *spp.	97.74% with *S. vinaceusdrappus*	MF356356	2.5
7K	Streptomyces enissocaesilis	100% with *S. enissocaesilis *and 100% with *S. rochei*	MF353986	0
7L	*Streptomyces *spp.	-	NA**	2.5
7M	*Streptomyces *spp.	97.32% with *S. niveoruber*	MF356357	0
7N	*Streptomyces *spp.	99.18% with *S. chromofuscus*	MF356358	5
7O	*Streptomyces *spp.	99.52% with *S. werraensis*	MF356359	6
8A	Streptomyces albogriseolus	99.79% with *S. albogriseolus*	MF353987	3
8B	Streptomyces albogriseolus	100% with *S. albogriseolus*	MF353988	0
8C	*Streptomyces *spp.	97.94% with *S. kebangsaanensis*	MF356360	4
8D	Streptomyces variabilis	99.90% with *S. variabilis*	MF353989	0
8E	Streptomyces werraensis	99.70% with *S. werraensis*	MF353990	0
8F	*Streptomyces *spp.	99.13% with *S. albogriseolus*	MF356361	1.33
8G	*Streptomyces* spp.	99.51% with *S. pomoeae*	MF356362	2.33
8H	Streptomyces albogriseolus	99.77% with *S. albogriseolus*	MF353991	0
8I	*Streptomyces *spp.	-	NA	0
8J	*Streptomyces *spp.	-	NA	1.67
8K	*Streptomyces *spp.	-	NA	2.29
8L	*Streptomyces *spp.	98.32% with *S. misionensis*	MF356363	3.33
8M	*Streptomyces *spp.	-	NA	2.22
8N	*Streptomyces* spp.	99.37% with *S. lomondensis* and 99.12% with *S. luteogriseus*	MF356364	1.33
8O	*Streptomyces *spp.	-	NA	4
8R	*Streptomyces *spp.	97.29% with *S. fumigatiscleroticus*	MF356365	4.17
10A	*Streptomyces *spp.	-	NA	0
10B	Streptomyces enissocaesilis	99.30% with *S. enissocaesilis *and 99.30 with *S. rochei*	MF353938	0
10C	Streptomyces werraensis	100% with *S. werraensis*	MF353939	1.2
10D	Streptomyces enissocaesilis	99.41% with *S. enissocaesilis *and 99.41% with *S. rochei*	MF353940	0
14A	*Streptomyces *spp.	-	NA	2
14B	*Streptomyces *spp.	97.80% with *S. fragilis*	MF356324	0
14C	*Streptomyces *spp.	96.83% with *S. leeuwenhoekii*	MF356325	0
14D	*Streptomyces *spp.	96. 32% with *S. leeuwenhoekii*	MF356326	0
14E	Streptomyces djakartensis	100% with *S. djakartensis*	MF353947	0
14F	*Streptomyces *spp.	-	NA	0
14G	*Streptomyces *spp.	96.52% with *S. hawaiiensis*	MF356327	0
14H	*Streptomyces *spp.	98.88% with *S. minutiscleroticus*	MF356328	3
14I	*Streptomyces *spp.	96.30% with *S. aurantiogriseus*	MF356329	2.5
14J	Streptomyces djakartensis	100% with *S. djakartensis*	MF353948	3
14K	Streptomyces djakartensis	100% with *S. djakartensis*	MF353949	0
19A	*Streptomyces *spp.	94.79% with *S. malachitofuscus*	MF356331	2.5
19B	*Streptomyces *spp.	98.54% with *S. minutiscleroticus*	MF353953	1.3
19C	*Streptomyces *spp.	-	NA	0
19D	*Streptomyces *spp.	99.55% with *S. werraensis*	MF356332	1.25
19E	*Streptomyces *spp.	94.91% with *S. werraensis*	MF356333	2
19F	*Streptomyces *spp.	-	NA	0
19G	*Streptomyces *spp.	-	NA	2.75
19H	Streptomyces griseostramineus	99.77% with *S. griseostramineus*, 99.77% with *Streptomyces *spp. SD528 (EU544233)	MF353954	6
19I	Streptomyces werraensis	100% with *S. werraensis*	MF353955	6.67
19J	*Streptomyces *spp.	99.33% with *S. werraensis*	MF356334	3
19K	*Streptomyces *spp.	95.56% with *S. thermocarboxydovorans*	MF356335	3.5
19L	*Streptomyces *spp.	-	NA	1.67
23A	*Streptomyces *spp.	-	NA	3.33
23B	Streptomyces fragilis	99.68% with *S. fragilis*	MF353961	1.6
29A	*Streptomyces *spp.	-	NA	2
29B	Streptomyces werraensis	99.88% with *S. werraensis*	MF353967	2.29
29C	Streptomyces cinnabarinus	98.48% with *S. cinnabarinus*	MF353968	2.75
29D	Streptomyces werraensis	99.55% with *S. werraensis*	MF353969	0

**Figure 3:**
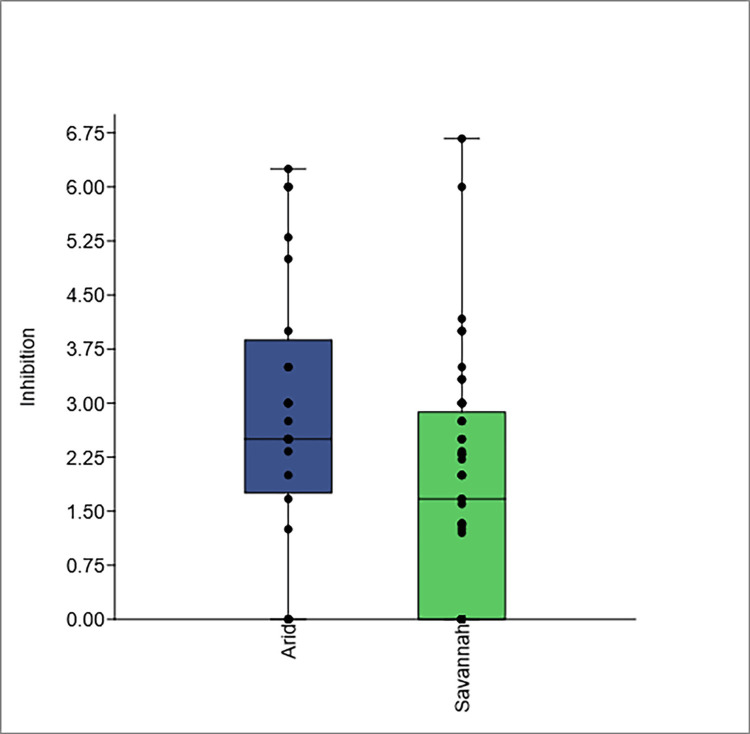
Box plot comparing inhibition values of soil Streptomyces spp. against Streptomyces sudanensis in arid (blue) and savannah (green) ecosystems. The arid ecosystem had a mean inhibition value of 2.8411 (N = 28) with a variance of 3.7218, while the savannah had a mean of 1.7139 (N = 49) and a variance of 2.7753. The difference in means was 1.1272, with a t-value of 2.6954 (p = 0.0086728) indicating statistical significance. Whiskers show the data range, and individual points indicate outliers. The inhibition value is the ratio of the inhibition zone (in mm) against *Streptomyces sudanensis* to the colony size of soil streptomycetes.

The inhibition zones for the identified soil *Streptomyces* strains varied significantly, with several strains demonstrating notable activity (Table 1, Figure 3). For instance, strain 7B showed an inhibition zone of 4 mm, while strains 7D and 7O exhibited larger zones of 6.25 mm and 6 mm, respectively. Strain 19H also demonstrated substantial inhibition with a zone of 6 mm. Several strains, including 7A, 7K, and 8B, showed no inhibition activity at all. Overall, these results highlight the diverse inhibitory capacities of the identified *Streptomyces* strains against the target organism.

Analysis of soil samples from arid and savannah ecosystems revealed significant differences in mean values through t-tests (Figure 3). The arid ecosystem had a mean of 2.8411 (N = 28) and a variance of 3.7218, while the savannah ecosystem had a mean of 1.7139 (N = 49) with a variance of 2.7753. The difference in means was 1.1272, with 95% confidence intervals indicating a statistically significant distinction. The t-test yielded a t-value of 2.6954 and a p-value of 0.0086728, providing strong evidence against the null hypothesis of equal means. With unequal variances, the t-value was 2.589 (p = 0.012585).

## Discussion

The diversity of actinomycetes, such as *Streptomyces* spp., is significantly influenced by a range of biotic and abiotic factors, including vegetation, soil type, and climate. Identification of how these factors shape microbial communities is essential for harnessing their ecological roles in agriculture and disease management, given their known pharmaceutical and biotechnological potential [10,11].

Our study successfully isolated *Streptomyces* using humic acid and ISP2 media, revealing notable phenotypic diversity. Microscopic images emphasize their characteristic filamentous structures, indicating broad functional capabilities [12]. Notably, Das et al. [13] have highlighted the underexplored potential of soil habitats in Northeast India for producing novel antimicrobial metabolites, addressing the challenge of antibiotic resistance.

The relationship between nutrient availability and microbial diversity is critical in savannah ecosystems, which exhibited lower inhibition values for *Streptomyces* [14]. Variations in grazing pressure can influence microbial populations and enzyme activities [15]. *Streptomyces* species adapt to nutrient-poor environments, enabling them to exert antagonistic effects against pathogens such as *S. sudanensis*. For example, the antimicrobial activity of *Streptomyces huasconensis* is linked to its production of diverse secondary metabolites [16].

Comparative analysis of inhibition values reveals significant ecological differences between arid and savannah ecosystems. In arid regions, higher average inhibition values (2.55 to 3.03) stem from high pH and low nutrient availability. In contrast, lower inhibition values in savannah ecosystems suggest a less specialized microbial community. Key statistical findings reinforce the conclusion that arid ecosystems harbor more effective *Streptomyces* strains for biological control. For instance, López-Reyes et al. [17] showed that *Streptomyces* sp. PR69 inhibited various plant pathogens, promoting plant growth.

Savannah ecosystems foster greater microbial growth due to favorable conditions, indicated by lower inhibition values. Enhanced nutrient availability and moisture lead to a more balanced microbial community, potentially reducing the dominance of *Streptomyces*. Furthermore, diverse plant species in these ecosystems can influence microbial interactions, affecting the ecological roles and antibiotic production of *Streptomyces* [18].

The genetic and morphological similarities between soil isolates and those linked to mycetoma cases underscore the ecological significance of certain *Streptomyces* strains in actinomycetoma epidemiology. While this study offers valuable insights, it has limitations. Conducted in specific regions of Sudan, findings may not be widely generalizable. A larger sample size could enhance statistical power, and focusing on additional environmental factors, such as microbial interactions and seasonal variations, is crucial. Isolation methods may not fully capture the diversity of *Streptomyces* strains, suggesting advanced techniques for future research. Lastly, while we primarily assessed antimicrobial properties against *S. sudanensis*, broader evaluations against other pathogens are needed to fully explore the biocontrol potential of *Streptomyces* spp.

## Conclusions

This study emphasizes the importance of soil properties in influencing the diversity and inhibitory potential of *Streptomyces* spp. against *S. sudanensis*. Our findings reveal the ecological significance of specific *Streptomyces* strains in actinomycetoma epidemiology and emphasize the need for further research on their genetic and morphological traits. Improving our understanding of these microbial communities may lead to novel biocontrol strategies for actinomycetoma and other soil-borne diseases, while also enhancing soil health in sub-Saharan Africa to boost agricultural productivity and ecosystem resilience.

## References

[1] Kämpfer P (2006). The family streptomycetaceae, part I: taxonomy. The Prokaryotes: A Handbook on the Biology of Bacteria.

[2] Seipke RF, Kaltenpoth M, Hutchings MI (2012). Streptomyces as symbionts: an emerging and widespread theme?. FEMS Microbiol Rev.

[3] Xie F, Pathom-Aree W (2021). Actinobacteria from desert: diversity and biotechnological applications. Front Microbiol.

[4] Lichon V, Khachemoune A (2006). Mycetoma : a review. Am J Clin Dermatol.

[5] Agarwal P, Jagati A, Rathod SP, Kalra K, Patel S, Chaudhari M (2021). Clinical features of mycetoma and the appropriate treatment options. Res Rep Trop Med.

[6] German DP, Weintraub MN, Grandy AS, Lauber CL, Rinkes ZL, Allison SD (2011). Optimization of hydrolytic and oxidative enzyme methods for ecosystem studies. Soil Biol Biochem.

[7] Hamid ME, Reitz T, Joseph MR (2020). Diversity and geographic distribution of soil streptomycetes with antagonistic potential against actinomycetoma-causing Streptomyces sudanensis in Sudan and South Sudan. BMC Microbiol.

[8] Rainey FA, Ward-Rainey N, Kroppenstedt RM, Stackebrandt E (1996). The genus Nocardiopsis represents a phylogenetically coherent taxon and a distinct actinomycete lineage: proposal of Nocardiopsaceae fam. nov. Int J Syst Bacteriol.

[9] Singh SK, Rai JP (2004). Soil microbial population and enzyme activity related to grazing pressure in alpine meadows of Nanda Devi Biosphere Reserve. J Environ Biol.

[10] Khan S, Srivastava S, Karnwal A, Malik T (2023). Streptomyces as a promising biological control agents for plant pathogens. Front Microbiol.

[11] Chouyia FE, Ventorino V, Pepe O (2022). Diversity, mechanisms and beneficial features of phosphate-solubilizing Streptomyces in sustainable agriculture: a review. Front Plant Sci.

[12] Dow L, Gallart M, Ramarajan M, Law SR, Thatcher LF (2023). Streptomyces and their specialised metabolites for phytopathogen control - comparative in vitro and in planta metabolic approaches. Front Plant Sci.

[13] Das R, Romi W, Das R, Sharma HK, Thakur D (2018). Antimicrobial potentiality of actinobacteria isolated from two microbiologically unexplored forest ecosystems of Northeast India. BMC Microbiol.

[14] Gebremedhn HH, Ndiaye O, Mensah S (2023). Grazing effects on vegetation dynamics in the savannah ecosystems of the Sahel. Ecol Process.

[15] Wen Y, Zhang G, Bahadur A (2022). Genomic investigation of desert Streptomyces huasconensis D23 reveals its environmental adaptability and antimicrobial activity. Microorganisms.

[16] Sharma P, Thakur D (2020). Antimicrobial biosynthetic potential and diversity of culturable soil actinobacteria from forest ecosystems of Northeast India. Sci Rep.

[17] López-Reyes PK, De la Torre-Zavala S, Cortés-González MM, Galán-Wong LJ, Avilés-Arnaut H (2024). Biological control of Streptomyces sp. PR69 against Phytophthora capsici and its growth-promoting effects on plants. Horticulturae.

[18] Barka EA, Vatsa P, Sanchez L (2016). Taxonomy, physiology, and natural products of actinobacteria. Microbiol Mol Biol Rev.

